# Nutrition update in gastric cancer surgery

**DOI:** 10.1002/ags3.12351

**Published:** 2020-06-08

**Authors:** Takeshi Kubota, Katsutoshi Shoda, Hirotaka Konishi, Kazuma Okamoto, Eigo Otsuji

**Affiliations:** ^1^ Division of Digestive Surgery Department of Surgery Kyoto Prefectural University of Medicine Kyoto Japan

**Keywords:** gastric cancer, nutrition, review, surgery

## Abstract

Patients with gastric cancer are often malnourished during tumor progression. Malnutrition is a risk factor for postoperative complications and a poor prognosis. Early evaluation and management of nutrition can improve these outcomes. Various combined indices in which albumin is the primary component are used to evaluate the nutritional status, including the Prognostic Nutritional Index, Glasgow Prognostic Score, and Controlling Nutritional Status score. Both the American Society for Parenteral and Enteral Nutrition and the European Society for Clinical Nutrition and Metabolism guidelines recommend immediate and early oral/enteral nutrition. However, few reports have described the additional effects of preoperative immunonutrition on clinical outcomes of gastric cancer surgery. Gastrectomy types and reconstruction methods that consider the postoperative nutritional status have been used when oncologically acceptable. Total gastrectomy has recently tended to be avoided because of its negative impact on nutritional status. New findings obtained from the emergence of continuous glucose measurement, such as glucose fluctuation and nocturnal hypoglycemia, may affect nutritional management after gastrectomy. Some prospective clinical studies on perioperative nutritional intervention have set postoperative body weight loss as a primary endpoint. It seems important to continue oral nutritional supplement, even in small doses, to reduce body weight loss after gastrectomy. Evidence generated by prospective, well‐developed randomized controlled studies must be disseminated so that nutritional therapy is widely recognized as an important multimodal therapy in patients undergoing gastric cancer surgery.

## Graphical abstract

Evaluating and addressing the issue of nutrition early can improve outcomes of gastric cancer surgery. For surgical procedures, gastrectomy types and reconstruction methods that consider postoperative nutritional status have been conducted. Evidence needs to be disseminated so that nutritional therapy is widely recognized as an important multimodal therapy for gastric cancer.

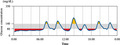

## 1 INTRODUCTION

Gastric cancer (GC) is the fifth most common type of cancer and the third most common cause of cancer‐related deaths worldwide.^1^ Despite continued developments in surgery and pharmacotherapy for GC, the prognosis generally remains poor. In Japan, GC surgery is improving with the advancement of instruments and an increased understanding of anatomy. Although no additional survival benefit has been demonstrated by excessive surgical invasion in prospective randomized clinical trials (RCTs),^2^, ^3^, ^4^ minimally invasive surgery including laparoscopic and function‐preserving surgery is expanding.

Malnutrition is reportedly a risk factor for postoperative complications and a poor prognosis.^5^ In addition to removing tumors, surgeons have begun to focus on perioperative nutritional management. For example, the concept of early recovery after surgery (ERAS), which was originally used for colorectal surgery,^6^ has now been applied to GC surgery. The effectiveness of the ERAS protocol in GC surgery (i.e., reducing complications, hospital stay, and cost) has been suggested. However, Yamagata et al^7^ warned that ERAS cannot achieve full penetration in Japan because most evidence is established in Western countries.

The aim of nutritional therapy is to improve the nutritional status, metabolism, incidence of postoperative complications, adherence to anticancer therapies, quality of life (QOL), and survival. In this review, we outline the current status and topics regarding perioperative nutritional management for GC surgery based on recent evidence.

## 2 NUTRITIONAL STATUS OF PATIENTS WITH GC

In patients with GC, malnutrition is caused by a decrease in food intake due to mechanical obstruction and cachexia, which occur during cancer progression. Cachexia is associated with tumor–host factors including tumor necrosis factor‐α, interleukin‐1, interleukin‐6, and leptin dysregulation. These factors may significantly influence appetite, muscle mass, and adipose tissues, leading to weight loss.^8^ Therefore, many patients with advanced GC often develop hypoproteinemia, dehydration, and electrolyte abnormalities. Nutritional evaluation is initially performed on all patients using the Subjective Global Assessment.^9^

### 2.1 Evaluation by biochemical factors

Albumin, rapid‐turnover proteins (prealbumin, transferrin, and retinal‐binding protein), C‐reactive protein, total cholesterol, cholinesterase, glucose, hemoglobin, neutrophils, and total lymphocytes are among the well‐known nutritional indicators monitored before GC surgery. Numerous studies have sought to develop more reliable, combined scoring systems that can identify patients with a poor nutritional status, such as the Prognostic Nutritional Index, Glasgow Prognostic Score, and Controlling Nutritional Status score. These systems have been used successfully to predict postoperative complications and survival.^10^, ^11^, ^12^ Table 1 shows combined indices that can reportedly be used to estimate the nutritional status relevant to short‐ and long‐term outcomes of GC surgery. While some of the algorithms are complicated, measurement of the serum albumin and lymphocyte levels is used more often as components of combined indices; they may still be the simplest, at‐a‐glance factors.

**TABLE 1:** Combined indices estimating nutritional status for gastric cancer surgery

Index	Components (the serum levels)
PNI	Albumin, lymphocyte
CONUT	Albumin, lymphocyte, total cholesterol
GPS	Albumin, C‐reactive protein
CRP/ALB ratio	Albumin, C‐reactive protein
NPS	Albumin, lymphocyte, neutrophil, monocyte, total cholesterol
SIS	Albumin, lymphocyte, monocyte
NLR	Neutrophil, lymphocyte
PLR	Platelet, lymphocyte
LMR	Lymphocyte, monocyte
TL score	Total cholesterol, lymphocyte

Abbreviations: CONUT, Controlling nutritional status; CRP/ALB, C‐reactive protein/albumin; GPS, Glasgow prognostic score; LMR, Lymphocyte/monocyte ratio; NLR, Neutrophil/lymphocyte ratio; NPS, Naples prognostic score; PLR, Platelet/lymphocyte ratio; PNI, Prognostic nutritional index; SIS, Systemic inflammatory score; TL score, Total cholesterol/lymphocyte score.

### 2.2 Evaluation by physical factors

Body weight (BW) loss before surgery, which is a simple nutritional index obtained from the Subjective Global Assessment, has long been used in clinical practice. In 1991, Haugstvedt et al^13^ reported that BW loss increased with age, with advanced stages of disease, in Lauren's diffuse vs intestinal tumor type, and with tumors located in the cardia. They further showed that increasing BW loss significantly reduced the resectability rate, but no association was found between preoperative BW loss and the postoperative complication rate.

Body mass index (BMI) is also a simple indicator of the physical condition, but it is paradoxical. Chen et al^14^ evaluated the morbidity and mortality risks in 1249 patients with GC undergoing gastrectomy based on their preoperative BMI (low, <18.5; normal, 18.5‐24.9; and high, >25.0 kg/m^2^). They found that a low BMI was associated with more severe postoperative complications and a poorer prognosis. Despite a higher risk of mild postoperative complication, patients with a high BMI exhibited paradoxically superior survival outcomes than patients with a normal BMI. Yang et al^15^ measured the visceral fat area (VFA) and evaluated the impact of obesity on postoperative complications as compared with the BMI. They found that VFA was an independent risk factor for postoperative complications and showed that VFA was superior to BMI in accurately and effectively predicting the impact of obesity on short‐term outcomes.

There is increasing evidence that as patients age, a relationship exists between sarcopenia and surgical outcomes. Sarcopenia is characterized by a loss of skeletal muscle mass and strength and is a major contributor to overall frailty. Sarcopenia is present in a large proportion of patients with advanced GC and significantly influences tolerance to chemotherapy, surgical complications, tumor recurrence, and survival.^16^ Moreover, one study showed that patients with a low skeletal muscle mass index had a higher age and significantly lower albumin level and BMI, indicating that skeletal muscle mass is correlated with the nutritional status of patients with GC.^17^ Many studies have shown that the preoperative skeletal muscle mass index is a useful nutritional determinant that predicts postoperative complications and survival after GC surgery.^18^ A preoperative exercise and nutritional support program have the potential to reduce sarcopenia and improve postoperative outcomes in advanced‐age patients with sarcopenia and GC.^19^

## 3 PREOPERATIVE NUTRITIONAL MANAGEMENT

As mentioned above, preoperative malnutrition may contribute to postoperative complications and a poor prognosis in patients with GC. Moreover, postoperative complications themselves can adversely affect the overall and recurrence‐free survival of patients with GC.^20^ Therefore, an appropriate assessment of the preoperative nutritional status through various biochemical and physiological tests and subsequent nutritional intervention before gastrectomy is essential for malnourished patients with GC.

### 3.1 Recommendation of oral/enteral nutrition

For malnourished patients with GC, peripheral parenteral nutrition or total parenteral nutrition (TPN) is often performed. While peripheral parenteral nutrition often does not provide enough energy or nutrients, TPN can provide sufficient amounts of nutrients for a long time. However, parenteral nutrition causes various impairments of host defense mechanisms, including gut immunity, systemic mucosal immunity, hepatic immunity, and peritoneal host defense.^21^ In addition, TPN requires a central vein catheter and is associated with more risks.

**FIGURE 1:**
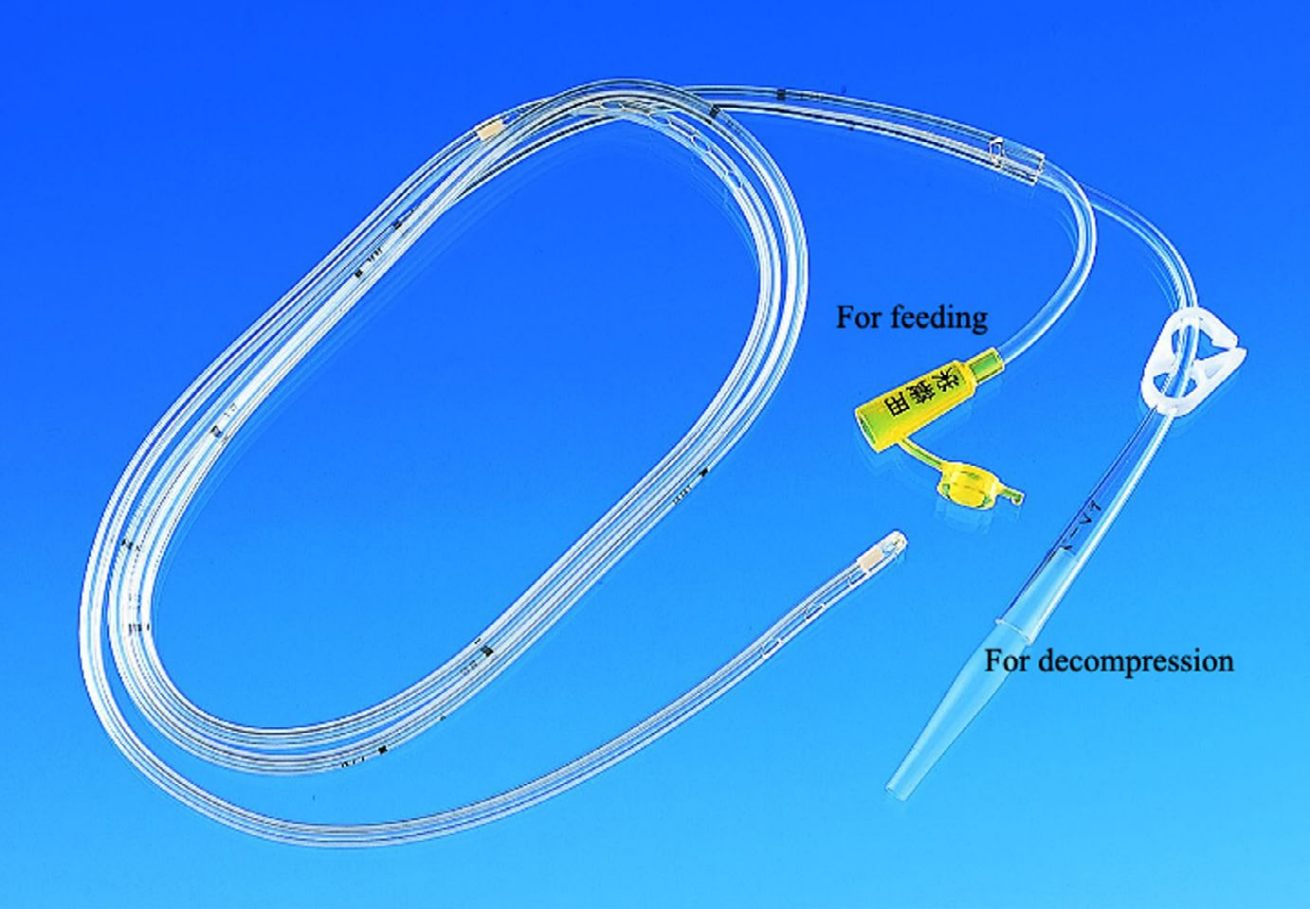
W‐ED^®^ tube (double elementary diet tube). The W‐ED^®^ tube (Covidien, Tokyo, Japan) is 150 cm long and has connecters for both drainage and nutrition. One lumen has its openings at the side of the tube, 60 cm above the leading edge, for decompression of the stomach. Another lumen has its openings at the end of the tube for feeding

Both the American Society for Parenteral and Enteral Nutrition (ASPEN) and the European Society for Clinical Nutrition and Metabolism (ESPEN) guidelines recommend oral/enteral feeding whenever possible.^22^, ^23^ Animal studies have demonstrated that fasting and malnutrition can result in intestinal mucosal atrophy and bacterial translocation.^24^ Although enteral atrophy is observed during fasting in humans, the change is minimal, and whether it is the result of bacterial translocation remains unclear.^25^ However, it appears that the immune barrier function is reduced.^26^ Therefore, patients should be treated with oral nutrition (ON) when possible; otherwise, enteral nutrition (EN) is the indicated administration route. In patients with pyloric stenosis with gastric dilatation, the leading edge of the enteral diet tube is placed beyond the stenosis using a fluoroscopy or endoscopy. It is possible to perform EN while decompressing the stomach using the double elementary diet tube (W‐ED^®^ tube; Covidien, Tokyo, Japan) (Figure 1). This tube can also be applied in the case of anastomotic leakage. Parenteral nutrition alone or with EN should be considered unless adequate nutrition can be administered by EN alone.

### 3.2 Immunonutrition

Many types of nutrients are used in enteral formulae (EF), each of which contains distinctive supplements. Therefore, they should be administered according to the nutritional status of each individual patient. In the 1990s, immunonutrition received significant attention after its usefulness was reported in various RCTs and meta‐analyses based on pharmacological effects.^27^ Both the ASPEN and ESPEN guidelines also recommended the administration of preoperative immunonutrients prior to cancer surgery.^28^, ^29^ Table 2 shows three studies from the 2000s that assessed the clinical outcomes of preoperative immunonutrition in patients with GC. Okamoto et al^30^ reported that preoperative oral administration of EF enriched with arginine, omega‐3 fatty acids, and RNA enhanced the patients’ immune status, reduced the duration of systemic inflammatory response syndrome, and decreased the incidence of postoperative infectious complications. However, the two subsequent studies did not demonstrate the usefulness of immunonutrition. Fujitani et al^31^ reported that 5‐day preoperative oral immunonutrition failed to provide any clear advantage in terms of early clinical outcomes or modification of the systemic acute‐phase response in well‐nourished patients with GC undergoing elective total gastrectomy (TG). Claudino et al^32^ retrospectively compared 164 patients with GC with or without immunonutrition and reported that preoperative immunonutrition did not reduce postoperative complications. Thus, the actual usefulness of immunonutrition remains controversial. However, no surgeons will object to providing any kind of nutritional intervention for malnourished patients with GC even for a limited period until surgery. The ERAS guideline describes the need to identify malnourished patients and to provide EN to these patients.^33^

**TABLE 2:** Effect of preoperative immunonutrition on gastric cancer patients with gastrectomy

Authors	Okamoto et al 2009	Fujitani et al 2012	Claudino et al 2020
Study design	RCT	RCT	Retrospective study
Sample size	60	244	164
Gastrectomy type	DG and TG	TG	DG and TG
Formula	Impact^®^	Impact^®^	Immune‐modulatory supplement^a^
Treatment period	7 d	5 d	5‐7 d
Endpoints	Rate of infectious postoperative complications Duration of SIRS	Rate of infectious postoperative complications Rate of surgical site infection	Rate of postoperative complications Length of hospital stay
Result	Positive	Negative	Negative

Note

Impact^®^ (Ajinomoto Pharmaceuticals, Japan).

Abbreviations: DG, distal gastrectomy; RCT, randomized controlled trial; SIRS, systemic inflammatory response syndrome; TG, total gastrectomy.

^a^ Oral or enteral, polymeric, hyperprotein diet, enriched with arginine, omega‐3 fatty acids, and nucleotides.

### 3.3 Nutrition support team

**FIGURE 2:**
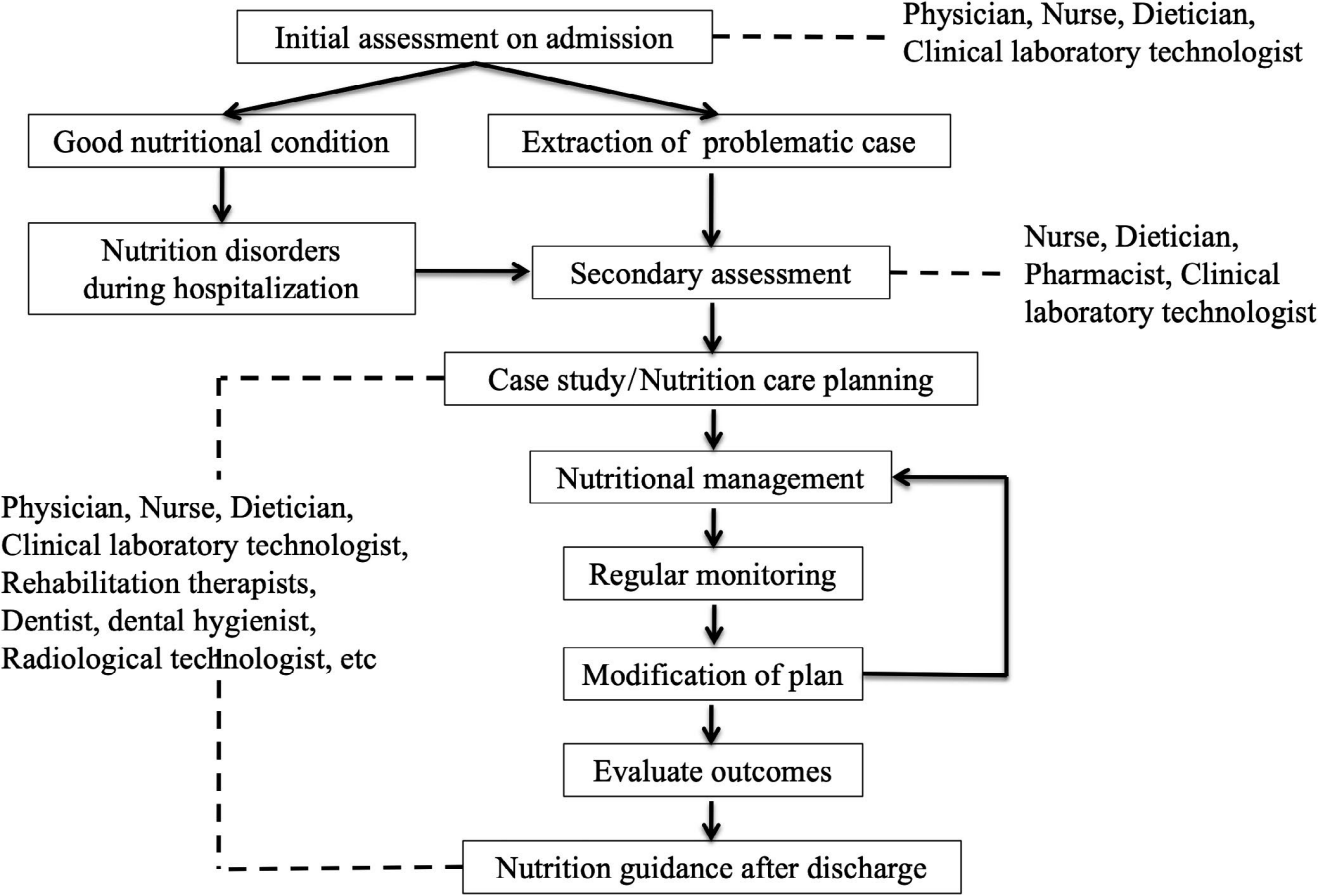
Nutrition support team (NST) activity flowchart. An NST consists of a physician, nurse, dietician, pharmacist, clinical laboratory technologist, rehabilitation therapists (speech‐language pathologist, physical therapist, occupational therapist), dentist, dental hygienist, radiological technologist. These team members provide nutrition‐related interventions to the patient through a multidisciplinary approach

In the 1960s, a nutrition support team (NST) was created, accompanying the development of TPN in the United States to manage patients who were malnourished or at risk for becoming malnourished by a multidisciplinary approach. An NST consists of a physician, nurse, dietician, pharmacist, clinical laboratory technologist, rehabilitation therapists (speech‐language pathologist, physical therapist, occupational therapist), dentist, dental hygienist, and radiological technologist, who are responsible for supporting all aspects of perioperative nutritional treatment. Some studies have confirmed that this multi‐professional NST provides nutritional care more effectively than team members acting independently.^34^ This innovation was adopted in Japan in the 2000s and has now become the gold standard for nutritional care in hospitals. Figure 2 shows a common NST activity flowchart with nutrition‐related intervention for a patient throughout the perioperative course.

## 4 IMPACT OF GASTRECTOMY TYPE ON NUTRITION

### 4.1 Minimally invasive, function‐preserving surgery

Gastrectomy disrupts the reservoir capacity, mechanical digestion, and gastric emptying. Because of the altered form and function of the stomach, various post‐gastrectomy syndromes can occur. In recent years, there has been a tendency to preserve the function of the stomach as much as possible while considering curability and age. Postoperative nutritional benefits of functional preservation have also been reported. Tsujiura et al^35^ confirmed that laparoscopic pylorus‐preserving gastrectomy is an acceptable and favorable operation for clinically diagnosed early GC with respect to long‐term survival and postoperative nutrition.

What about the impact of different approaches, such as laparoscopic gastrectomy or open gastrectomy, on the postoperative nutritional status? Laparoscopic surgery is expected to have nutritional benefits such as early recovery with less pain, less frequent use of analgesics, early mobilization, early recovery of intestinal peristalsis, and a shorter hospital stay. However, there appears to be no difference in the postoperative short‐term nutritional status, including BW loss, which may be nutritionally related to a worse prognosis because of differences in surgical approaches.^36^ Aoyama et al^36^ suggested that the level of surgical stress (white blood cell count and interleukin‐6 level) and the preoperative nutritional status were similar between laparoscopic and open distal gastrectomy, excluding patients who developed morbidities.

### 4.2 Avoidance of TG

TG has many potential disadvantages, especially in terms of the hematological and nutritional status, that result in severe BW loss and decreased physical activity. Therefore, attempts to avoid TG, especially in advanced‐age or high‐risk patients, have been made to minimize postoperative malnutrition and BW loss when oncologically acceptable.

With the increase of upper GC cases in Japan, the ratio of proximal gastrectomy and subtotal gastrectomy with a small stomach remnant has increased. Proximal gastrectomy was once unfavorable because of problems such as reflux esophagitis and stenosis. In particular, reflux interferes with oral intake because of heartburn and vomiting. In recent years, however, proximal gastrectomy has been performed without hesitation even for advanced‐age patients because the reconstruction procedures, with some modifications for preventing reflux, have become consistent.^37^ Additionally, Furukawa et al^38^ reported that laparoscopic subtotal gastrectomy with a very small stomach remnant had more favorable short‐term outcomes and nutritional status than laparoscopic total and proximal gastrectomy. They considered that the remaining stomach, although very small, maintains ghrelin secretion and reduces reflux through cardia preservation, contributing to a favorable postoperative nutritional status.

## 5 POSTOPERATIVE NUTRITIONAL CARE

### 5.1 Early postoperative dietary management

Early initiation of ON or EN after gastrectomy has recently been recommended. This may be due to the “no fasting” element of ERAS. The ERAS consensus guidelines recommend offering patients drink and food at will from 1 day after TG.^33^ In contrast, the Japanese Gastric Cancer Treatment Guideline ver.5 states that drink should be offered after postoperative day 1 and that a solid diet should begin from postoperative day 2 to 4 regardless of the gastrectomy type.^39^ The initiation of ON in the Japanese guideline seems slightly slower than that in Europe. Many surgeons may be reluctant to initiate early ON after TG. Sierzega et al^40^ reported the safety and feasibility of early postoperative ON even after gastrectomy, including TG. In fact, there is no report that has stated that early ON increased any adverse events, including anastomotic leakage. Conversely, a Japanese multicenter RCT in 2018 showed that early ON did not shorten the postoperative hospital stay after distal gastrectomy.^41^ Thus, the benefits of early ON after gastrectomy require further investigation.

Placement of a jejunostomy feeding tube should be considered if ON is likely to be impaired, such as in advanced‐age patients undergoing TG, patients with severe preoperative malnutrition, patients who are expected to lose BW after surgery, and patients at high risk for postoperative complications. Early initiation of EN can ensure postoperative nutritional management. Some reports have shown that EN contributes to early recovery of intestinal function and postoperative nutritional status, a reduction in postoperative complications, a shortened hospital stay, and immune function in patients with GC.^42^ EN may be effective because, unlike ON, it can be forcibly administered regardless of patient preference.

### 5.2 Clinical studies of postoperative oral nutritional supplement (ONS)

Adjuvant S‐1 chemotherapy is the standard treatment for patients with stage II or III GC in Japan.^39^ Aoyama et al^43^ reported that severe BW loss, which is closely associated with poor S‐1 compliance, is an important risk factor for survival of patients with stage II or III GC who have undergone gastrectomy. Conversely, Yamashita et al^44^ reported that BW loss did not affect S‐1 compliance in their multicenter study of a larger number of patients with GC. Therefore, the association between BW loss and postoperative S‐1 compliance is controversial. In some prospective clinical studies on perioperative nutritional intervention, BW loss was set as a primary endpoint (Table 3). Ida et al^45^ could not demonstrate the efficacy of an eicosapentaenoic acid‐enriched ONS, consisting of 600 kcal for 7 days before and 21 days after surgery, on BW loss after TG for GC compared with a regular diet. They concluded that the negative result was due to decreased oral intake of a regular diet by adding the ONS. In contrast, Kobayashi et al^46^ administered 400 kcal/d of ONS containing major nutrients, vitamins, minerals, and trace elements within 7 days postoperatively and continued the supplement for 3 months postoperatively. They found a significant reduction in BW loss for patients who tolerated >200 kcal/d compared with those who could not tolerate this amount. Kimura et al^47^ reported that administration of 300 kcal/d of an elemental diet containing essential amino acids and a low‐fat content for 6 to 8 weeks in the early post‐gastrectomy period reduced BW loss not only at postoperative 6 to 8 weeks but also at 1 year in patients who underwent TG. These results suggest that it is important to continue ONS even in small doses to reduce BW loss after gastrectomy.

**TABLE 3:** Effect of postoperative ONS on BW loss reduction after gastrectomy – Recent prospective clinical studies in Japan

Authors	Ida et al, 2017	Kobayashi et al, 2017	Kimura Y et al, 2019
Study design	RCT	Prospective study	RCT
Sample size	123	82	106
Gastrectomy type	TG	TG	TG and DG
Formula	ProSure^®^	Racol® NF	Elental^®^
Calorie (kcal/d)	600	400	300
Days preoperatively	7	0	0
Days postoperatively	21	90	42‐56
Effect on BW loss reduction	Negative	Positive	Positive^a^

Note

ProSure^®^ (Abbott Laboratories, UK). Racol® NF (Otsuka Pharmaceutical Factory, Japan). Elental^®^ (Ajinomoto Pharmaceuticals, Japan).

Abbreviations: BW, body weight; DG, distal gastrectomy; ONS, oral nutritional supplement; RCT, randomized controlled study; TG, total gastrectomy.

^a^ Positive only in patients who underwent TG.

The placement of a feeding jejunostomy tube for nutritionally high‐risk patients is one of the strategies mentioned above. Baker et al^48^ suggested that home EN for 6 weeks through a feeding jejunostomy tube did not affect oral intake of a regular diet and improved postoperative nutrition following TG.

### 5.3 Perioperative hyperglycemia

Even patients without diabetes may develop hyperglycemia after surgery, and postoperative hyperglycemia increases the occurrence of a multitude of adverse clinical outcomes including surgical site infection (SSI), pneumonia, sepsis, cardiovascular complications, and acute kidney injury.^49^ Surgical stress activates the sympathetic nervous system and causes increased secretion of catecholamines, cortisol, growth hormone, glucagon, and other factors. These counter‐regulatory hormones increase hepatic glucose production, promote gluconeogenesis,^50^ and interfere with peripheral glucose uptake to create a state of relative insulin resistance,^51^ resulting in hyperglycemia. In ERAS, blood glucose management was not initially a recommended item, but it was added as a recommended item in the guidelines published in 2012.^52^

In 2001, Van den Berghe et al^53^ reported the benefit of intensive insulin therapy (IIT) to control the blood glucose at 80 to 110 mg/dL, primarily in patients undergoing cardiac surgery. However, it was later reported that IIT resulted in a high frequency of hypoglycemia and was less beneficial for patients with diabetes mellitus.^54^ Therefore, IIT should not be a standard postoperative management. From the perspective of SSI prevention, Takesue and Tsuchida^55^ reported that a target blood glucose level of ≤ 150 mg/dL is recommended in patients without diabetes who undergo gastroenterological surgery. The study included 360 patients who underwent gastrectomy out of 1555 patients, and given that the cause of hyperglycemia is surgical stress, this criterion can be adequately applied to patients with GC. The American College of Surgeons and Surgical Infection Society also published a consensus report indicating that better short‐term perioperative glucose control (110‐150 mg/dL) is important for all patients to lower the SSI risk.^56^

### 5.4 Hypoglycemia as a post‐gastrectomy syndrome

**FIGURE 3:**
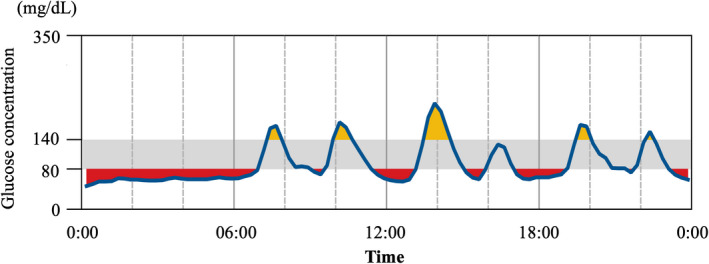
Typical daily glucose profile of a patient with gastric cancer undergoing total gastrectomy. This profile shows the trend obtained by continuous glucose monitoring for a patient undergoing total gastrectomy in our hospital. The profile shows prominent glucose fluctuation with postprandial and nocturnal hypoglycemia. Red and yellow indicate the area below and above the target glucose range (80‐140 mg/dL), respectively

Late dumping syndrome is a well‐known post‐gastrectomy syndrome that negatively affects patient QOL by causing hypoglycemia secondary to excess insulin secretion following meal‐induced hyperglycemia. With the recent emergence of continuous glucose monitoring, it has become apparent that patients with gastrectomy have a higher frequency of hypoglycemia and glucose fluctuation than expected.^57^ This suggests that the formerly known “dumping syndrome” appears to explain only a fraction of the postoperative glucose fluctuations present during the course of a day. In Figure 3, the glucose profile in one of our patients who underwent TG indicated that postprandial hypoglycemia and nocturnal hypoglycemia were evident throughout the day. Glycemic variability and hypoglycemia are known to have adverse effects on cardiovascular events and cognitive dysfunction.^58^, ^59^

## 6 CONCLUSION AND FUTURE PROSPECTS

This review summarizes the current status and various nutritional issues in GC surgery. The primary endpoint of GC surgery is to improve survival, and the role of nutritional treatment is to provide support during the perioperative period while maintaining patient QOL. Direct evidence is difficult to obtain in the area of nutrition. Evidence generated by prospective, well‐developed RCTs must be disseminated so that nutritional therapy is widely recognized as a multimodal therapy for GC.

## DISCLOSURE

Conflict of Interest: The authors declare no conflicts of interest for this article.
